# Montelukast and Acute Coronary Syndrome: The Endowed Drug

**DOI:** 10.3390/ph15091147

**Published:** 2022-09-14

**Authors:** Basil Mohammed Alomair, Hayder M. Al-kuraishy, Ali I. Al-Gareeb, Sadiq M. Al-Hamash, Michel De Waard, Jean-Marc Sabatier, Hebatallah M. Saad, Gaber El-Saber Batiha

**Affiliations:** 1 Internal Medicine, Endocrinology and Diabetes, Department of Medicine, College of Medicine, Aljouf University, Sakaka 72388, Saudi Arabia; 2 Department of Pharmacology and Toxicology, College of Medicine, Al-Mustansiriyah University, Baghdad P.O. Box 14132, Iraq; 3 Smartox Biotechnology, 6 Rue des Platanes, 38120 Saint-Egrève, France; 4 L’institut du Thorax, INSERM, CNRS, Université de Nantes, 44007 Nantes, France; 5 LabEx «Ion Channels, Science & Therapeutics», Université de Nice Sophia-Antipolis, 06560 Valbonne, France; 6 Institut de Neurophysiopathologie (INP), Faculté des Sciences Médicales et Paramédicales, Aix-Marseille Université, CNRS UMR 7051, 27 Bd Jean Moulin, 13005 Marseille, France; 7 Department of Pathology, Faculty of Veterinary Medicine, Matrouh University, Marsa Matruh 51744, Egypt; 8 Department of Pharmacology and Therapeutics, Faculty of Veterinary Medicine, Damanhour University, Damanhour 22511, Egypt

**Keywords:** acute coronary syndrome, leukotriene, montelukast, antileukotrienes therapy

## Abstract

Acute coronary syndrome (ACS) is a set of signs and symptoms caused by a reduction of coronary blood flow with subsequent myocardial ischemia. ACS is associated with activation of the leukotriene (LT) pathway with subsequent releases of various LTs, including LTB4, LTC4, and LTD4, which cause inflammatory changes and induction of immunothrombosis. LTs through cysteine leukotriene (CysLT) induce activation of platelets and clotting factors with succeeding coronary thrombosis. CysLT receptor (CysLTR) antagonists such as montelukast (MK) may reduce the risk of the development of ACS and associated complications through suppression of the activation of platelet and clotting factors. Thus, this critical review aimed to elucidate the possible protective role of MK in the management of ACS. The LT pathway is implicated in the pathogenesis of atherosclerosis, cardiac hypertrophy, and heart failure. Inhibition of the LT pathway and CysL1TR by MK might be effective in preventing cardiovascular complications. MK could be an effective novel therapy in the management of ACS through inhibition of pro-inflammatory CysLT1R and modulation of inflammatory signaling pathways. MK can attenuate thrombotic events by inhibiting platelet activation and clotting factors that are activated during the development of ACS. In conclusion, MK could be an effective agent in reducing the severity of ACS and associated complications. Experimental, preclinical, and clinical studies are recommended to confirm the potential therapeutic of MK in the management of ACS.

## 1. Introduction

Acute coronary syndrome (ACS) is a set of signs and symptoms caused by a reduction of coronary blood flow with subsequent myocardial ischemia [1]. According to the electrocardiographic (ECG) changes and duration of symptoms, ACS is divided into unstable angina, which represents 38%; ST-elevation myocardial infarction (STEMI) representing 30%, and non-ST-elevation myocardial infarction (NSTEMI) representing 25%. Unstable angina of ACS is differentiated from stable angina, as it occurs suddenly at rest with little response to treatment [2].

The cardinal clinical features of ACS are chest pain, dyspnea, nausea, vomiting, sweating, and tachycardia [3]. ACS is mainly caused by coronary thrombosis, coronary vasospasm, aortic valve stenosis, severe anemia, and pulmonary hypertension [4]. The underlying pathogenic mechanisms of ACS are related to rupture of atheroma in 60% and atheroma erosion in 30%. Most of the atheroma rupture induces the development of STEMI, while the development of atheroma erosion induces propagation of NSTEMI [5].

Plague rupture provokes in situ inflammatory reactions and thrombosis with subsequent ischemic-reperfusion injury (IRI). Thrombolytic therapy is indicated for STEMI, while nitroglycerin, antiplatelet, low-molecular-weight heparin (LMWH) such as enoxaparin, and heparin-like drugs such as fondaparinux are effective in this state [6,7].

It has been reported that leukotriene receptor (LT) receptor antagonists such as montelukast (MK) may have potential beneficial effects in the cardiovascular field [8]. Thus, this critical review aimed to elucidate the possible protective role of MK in the management of ACS.

## 2. Leukotriene Pathway

**Figure 1:**
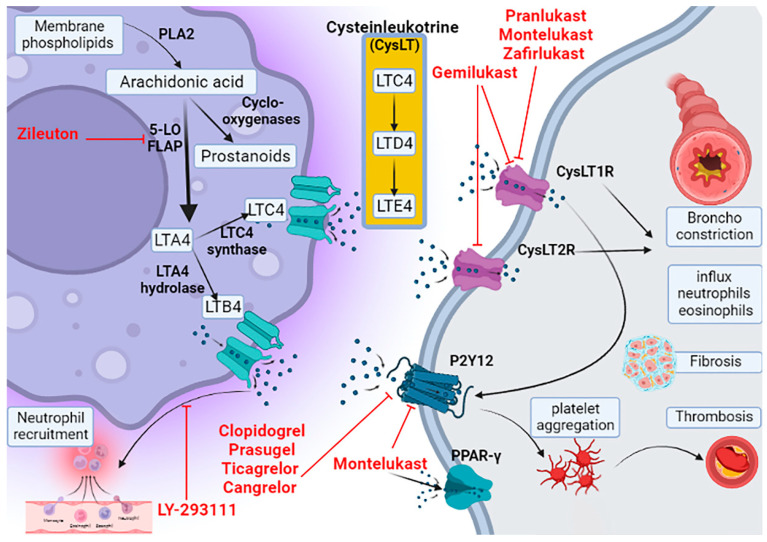
Leukotriene pathway and its inhibitors: Membrane phospholipids hydrolyzed by phospholipase A to arachidonic acid (AA), which, by the action of 5-lipoxygenase (5-LO), convert to leukotriene A4 (LTA4). Further, LTA4 is converted to LTB4 by the action of LTA4 hydrolase and to cysteinleukotriene (CysLT) by the action of LTC4 synthase. CysLTs act on CysLT receptors (CysLT1R and CysLT2R).

The LT name was initially described in 1979 by Swedish Bengt Samuelsson as a slow-reaction, smooth-muscle-stimulating substance that was introduced by Feldberg and Kellaway between 1938 and 1940 [9]. LTs are inflammatory eicosanoid mediators generated by leukocytes through oxidation of arachidonic acid (AA) and eicosapentaenoic acid (EPA) by the action of 5-lipoxygenase (5-LO) [9]. LTs use lipid signaling to express and convey information in autocrine and paracrine patterns to control immune functions. Usually, the production of LTs is correlated with the release of prostaglandins and histamines [10]. By the action of 5LO or 5LO-activating protein (FLAP), the AA is converted to LTA4, which is converted through LTA4 hydrolase and LTC4 synthase to LTB4 and cysteinleukotriene (CysLT), respectively. LTB4 is inhibited by LY-293111 a specific inhibitor of the LTB4 receptor, while MK, pranlukast, and zafirlukast block CysLT receptor (CysLTR) type 1 [11] (Figure 1).

Thus, there are two types of LTs, namely LTB4 and CysLT, which include LTC4, LTE4, and LTD4. CysLTs are involved in the development of allergy and anaphylaxis. Specifically, LTB4 induces recruitment of neutrophils with induction of tissue injury and release of pro-inflammatory cytokines. Therefore, LTB4 antagonists inhibit the development and propagation of neutrophil-mediated disorders [12,13]. LTA4 is mainly converted to LTB4 and has few inflammatory effects. LTC4 is mainly expressed by eosinophil and mast cells, which are converted outside the cells to form LTD4 and LTE4, which have inflammatory effects. Moreover, LTG4 is regarded as an active metabolite of LTE4 that has unknown biological effects [14,15].

There are two main types of CysLT receptors, which are CysLTR1 and CysLTR2. CysLTR1 is a G-protein coupled receptor that is mainly activated by LTD4 and LTC4, causing airway bronchoconstriction and increasing vascular permeability, the influx of neutrophils and eosinophils, fibrosis and collagen deposition, and membrane hypertrophy of respiratory epithelium [16]. Additionally, activation of CysLTR1 is associated with disruption of the blood–brain barrier (BBB) with induction of neuroinflammation and convulsion. Therefore, CysLTR1 antagonists could be effective against the development of Parkinson’s disease, Alzheimer’s disease, encephalomyelitis, and multiple sclerosis [17]. CysLTR1 is inhibited by MK, pranlukast, and zafirlukast [17]. CysLTR2 is activated by LTD4 and LTC4, and it is involved in the development of hypersensitivity and allergic reactions [18,19]. CysLTR2 is chiefly expressed in the platelets, eosinophils, macrophages, and mast cells. Furthermore, CysLTR2 is highly expressed in the heart, adrenal gland, endothelial cells, smooth muscle cells, and airway epithelial cells [18]. MK and other CysLTs only block CysLTR1; however, gemilukast inhibits both CysLTR1 and CysLTR2, which can be effective in the management of asthma [20]. Other types of LT receptors such as GPR99, which is called oxoglutarate or CysLTR3, are stimulated by LTE4 and α-ketoglutarate, while GPR99 is inhibited by MK [21]. Overexpression of GPR99 is linked with the development of hypertension [21].

GPR17 is a G-protein coupled receptor stimulated by LTC4, LTD4, and uracil nucleotides. GPR17 chemical structure is intermediate between CysLTR1 and CysLTR2. GPR17 is highly distributed in the central nervous system (CNS) mainly in the microglia cells [22]. Cangrelor, which is a purinergic receptor (P2Y) antagonist, blocks GPR17 [23].

Furthermore, P2Y12 is activated following activation of CysLTR1 by LTs; therefore, inhibition of P2Y12 by a specific antagonist attenuates the CysLTR1-dependent effect of LTs. P2Y12 is a chemoreceptor stimulated by adenosine diphosphate (ADP) and belongs to G-protein coupled purinergic receptor group [24]. P2Y12 is involved in platelet aggregation and thrombosis; it is inhibited by antiplatelet drugs such as clopidogrel, prasugrel, ticagrelor, and cangrelor [24]. Notably, P2Y12 is also expressed in the vascular smooth muscle and immune cells and involved in the pathogenesis of inflammation through the promotion of the interaction between platelets and leukocytes with the release of inflammatory cytokines [24]. Kang et al. illustrated that MK attenuates bone loss through inhibition expression of P2Y12 [25]. Moreover, LTs can activate peroxisome proliferator-activated receptors (PPAR). Interestingly, zafirlukast and MK are regarded as a potent modulators of PPAR-γ [26]. LTs are intricate in the pathogenesis of asthma, dementia, and cognitive dysfunction [26].

These findings suggest that LTs through activation of LTRs and other related receptors may be involved in the pathogenesis of respiratory and systemic disorders.

## 3. Pharmacology of Montelukast

**Figure 2:**
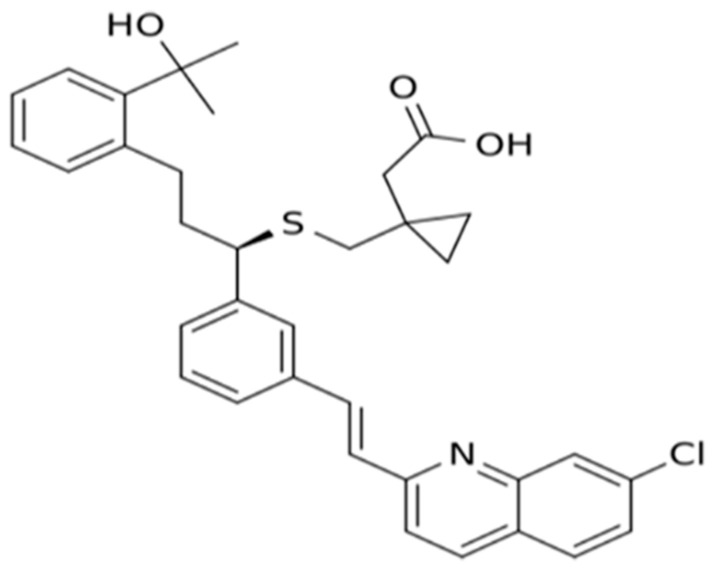
Chemical structure of montelukast (Reprinted from Ref. [27]).

MK is a monocarboxylic acid with aliphatic sulfide from the quinolone family (Figure 2).

MK is an orally active CysLTR1 antagonist that blocks the action of LTD4 used in the prophylaxis of asthma and management of various inflammatory disorders [28]. Similarly, MK is indicated in the management of urticarial and allergic rhinitis [28]. MK was approved by the Food and Drug Administration (FDA) in 1998 in the USA. The use of MK is associated with the development of many adverse effects, including vomiting, nausea, skin rash, angioedema, mild fever, malaise, paresthesia, muscle cramps, and seizure [28,29]. Prolonged use of MK is linked with the development of neuropsychiatric disorders, including nightmares, insomnia, depression, anxiety, and aggression [29]. In 2009, the FDA declared that long-term use of MK was associated with increased risk of suicidal behavior [29]. In 2020, the FDA put MK in a boxed warning about the risk of neuropsychiatric disorders and advises to limit the use of MK for simple allergic reactions when safe alternatives are available [30].

MK is rapidly absorbed from the intestine with 64% bioavailability, and its absorption is not affected by food. MK plasma concentration is attained within 2–4 h with high plasma protein binding. MK has few drug–drug interactions though it inhibits liver metabolizing enzyme CYP2C8. MK and its metabolites are highly distributed and excreted by bile [31]. The lethal dose in 50% (LD50) of MK is more than 5000 mg/kg [31].

## 4. Montelukast and Cardiovascular Complications

The general expression of CysLTR is associated with the propagation of different cardiovascular complications [32]. CysLT2R is highly expressed in the human atrium, ventricles, apex, septum, and Purkinje cells. In addition, CysLT2R is expressed on the endothelial cells, monocytes, and smooth muscle cells, while CysLT1R is chiefly present in the macrophages and monocytes [32]. Surprisingly, only CysLT2R is expressed in coronary smooth muscles, whereas aortic smooth muscles highly expressed CysLT1R [32]. Of note, CysLTR exerts negative inotropic effects and reduces coronary blood flow without chronotropic effects [33].

**Figure 3:**
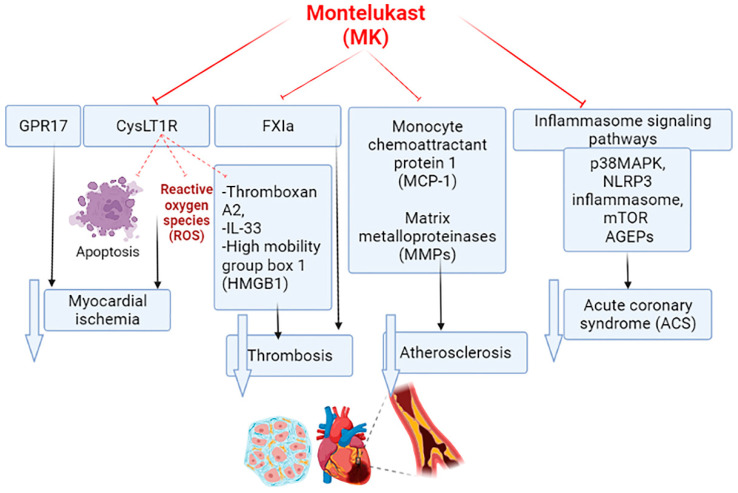
Mechanisms of montelukast in different cardiac and vascular diseases: Montelukast (MK) inhibits inflammasome signaling pathways, including p38 mitogen-activated protein kinase (p38MAPK), nod-like receptor pyrin 3 (NLRP3) inflammasome, mechanistic target of rapamycin (mTOR), and advanced glycation end-products (AGEPs). In addition, MK attenuates activation of cysteinleukotrine (CysLT) and activated factorXI with subsequent inhibition of inflammation and thrombosis-induced myocardial infarction and acute coronary syndrome (ACS).

MK and other CysLTR antagonists were originally developed for the management of asthma. In virtue of its anti-inflammatory property, MK could be effective in treating atherosclerosis and metabolic syndrome [34]. It has been shown that asthmatic patients on MK treatments showed a low cardiovascular risk index since MK decreases the risk of stroke, myocardial infarction, and other cardiovascular complications [8]. Of interest, MK inhibits epoxide hydrolase, which hydrolyzes beneficial epoxyeicosatrienoic acid to inactive dihydroxyeicosatrienoic acid [35]. Thus, MK and other CysLTR antagonists through activation of PPAR-γ and inhibition of epoxide hydrolase may reduce cardiovascular complications through the improvement of endothelial function and insulin sensitivity [36]. Animal model studies revealed that MK reduced the risk for the development of myocardial infarction, ischemic reperfusion injury, and atherosclerosis [37,38]. Ge et al. observed that MK had an anti-atherogenic effect by inhibiting the expression of monocyte chemoattractant protein 1 (MCP-1) in rabbits with induced carotid injury [38]. Likewise, Liu’s experimental study confirmed that MK reduced the propagation of atherosclerosis by inhibiting the expression of matrix metalloproteinases (MMPs) (Figure 3) [39]. Activation of PPAR-γ and inhibition of soluble epoxide hydrolase by MK attenuates propagation of oxidative stress-induced endothelial injury through suppression expression of AA metabolites [38]. Wang and colleagues observed that over-expression of soluble epoxide hydrolase was engaged with the development of atherosclerosis by reducing the anti-inflammatory anti-thrombotic effects of epoxyeicosatrienoic acid [40].

In the atherosclerotic lesions, the expression of 5-LO and FLAP and their metabolites, including LTB4, LTA4, and LTC4 and LTD4 and LTC4 are increased with the augmentation of the expression of CysLTR [41]. These findings suggest close relationships between the development of atherosclerosis and the activation of LT pathway, so targeting this pathway could limit the progression of the atherosclerotic process. In atherosclerotic lesions, the activated macrophages are augmented, which are the major source of 5-LO [41]. A clinical study observed that long-term treatment with MK reduced serum level of C-reactive protein (CRP) in the asthmatic patients [42]. Long-term follow-up studies proposed that MK by its anti-inflammatory effect may inhibit the advancement of atherosclerosis [43]. It has been shown that LT signaling pathway is implicated in the pathogenesis of atherosclerosis through suppression of lipid retention, accumulation of foam cells, and intimal hyperplasia with subsequent propagation of atherosclerosis [43].

Furthermore, LT increases the risk for rupture of atherosclerotic plaque through degradation of extracellular matrix [43]. For these reasons, augmentation of LTs may be correlated with the development of ischemic changes. In addition, over-expression of CysLTR in the atherosclerotic lesions increases the risk of atherosclerotic complications such as myocardial infarction, ischemic stroke, and aortic aneurysm [44]. Deletion of 5-LO in experimental mice reduced the development of atherosclerotic lesions by about 25-fold [45]. Likewise, an experimental study demonstrated that atorvastatin inhibits the advancement of atherosclerotic lesions by inhibiting the expression of CysLT1R and FLAP as well as regulating the expression of 5-LO [46].

Regarding clinical studies, Revan et al. found that genetic variation in the LT pathway was linked with the development of early atherosclerotic lesions [47]. Genetic variations in the expression of 5-LO are associated with increasing intima-media thickness and progression of atherosclerosis in a cohort study [48]. These verdicts implicated the LT pathway in the pathogenesis of atherosclerosis; hence, LT pathway inhibitors or CysLTR antagonists could be effective in preventing the development of atherosclerosis. Different studies revealed that CysLT1R antagonists such as MK and FLAP inhibitors decrease the development of atherosclerosis in mice [49,50].

In the atherosclerotic lesions, LTC4 expression and its mediator, known as multi-drug resistance protein 1 (MRP-1), were increased, causing oxidative stress injury in humans [51]. Of interest, LTC4 and LTD4 increase the expression of adhesion molecules such as P-selectin in the CysLT2R-dependent pathway, and thus, MK may be ineffective in the suppression of this pathway, as it merely blocks CysLT1R [52]. Furthermore, the induction release of pro-inflammatory cytokines and chemokines by LTD4 was not suppressed by the action of MK and other CysL1TR antagonists, as the release of inflammatory cytokines are mainly mediated by the CysL2TR [53]. Remarkably, LTD4 can induce the release of anti-inflammatory cytokine IL-10, which has a protective immunomodulatory effect against the development of atherosclerosis [53].

Moreover, overexpression of GPR99 is associated with the development of cardiac hypertrophy [54]. Nguyen et al. illustrated that MK can inhibit the pharmacological activity of GPR99, thereby limiting inflammatory progression and development of cardiac hypertrophy [55]. Further, changes in the expression of GPR107 and GPR99 could be the possible cause for the propagation and pathophysiology of hypertension in rats [21]. Moreover, the development and progression of chronic heart failure are associated with the escalation of the LT pathway [56]. Inhibition of LTB4 may reduce the inflammatory changes and progression of heart failure [56]. Suppression of LTB4 and AA metabolites decreases the risk for the development of heart failure [56].

These observations suggest that the LT pathway is implicated in the pathogenesis of atherosclerosis, cardiac hypertrophy, and heart failure. Inhibition of the LT pathway and CysL1TR by MK might be effective in preventing cardiovascular complications.

## 5. Montelukast and Acute Coronary Syndrome

Notably, soluble epoxide hydrolase increases the expression of fatty acid synthase in the mononuclear cells in patients with ACS [57]. A prospective study involving 65 patients with ACS compared to 65 healthy controls illustrated that serum levels of soluble epoxide hydrolase and fatty acid synthase were increased in ACS patients [57]. A study including 667 ACS patients revealed that high-soluble epoxide hydrolase serum level is correlated with the severity of ACS [58]. Suppression of soluble epoxide hydrolase attenuates cardiac ischemic-reperfusion injury in vivo [59]. In addition, inhibition of soluble epoxide hydrolase attenuates the progression of hypertension, which is the major risk factor for the development of ACS [59]. These findings suggest that inhibition of soluble epoxide hydrolase by MK could be a therapeutic strategy for preventing the development of ACS.

On the other hand, PPAR-γ agonists were reported to be effective in the reduction of ACS [60]. PPAR-γ agonists reduce the expression of pro-inflammatory cytokines and macrophage activation in the coronary bed [60]. In a case-control study, PPAR-γ serum level was reduced in ACS patients and inversely correlated with cardiac troponin compared with controls [61]. Therefore, PPAR-γ serum level could be a predictive biomarker for the development of ACS mainly myocardial infarction [61]. Single-gene polymorphism in PPAR-γ predisposes the development of ACS in the Turkish population [62]. Remarkably, expression of LTC4 and CysLT1R are increased during adipogenesis through upregulation of PPAR-γ [63]. Therefore, inhibition of CysLT1R by MK might be effective in the management of obesity-induced inflammatory disorders.

Of note, LTC4 increases intima-media thickness and calcium contents in the coronary atherosclerotic plaques [64]. In addition, hypoxic stress in ACS increases the expression of CysLT1R and LTC4 in mice [49]. Within coronary atherosclerotic lesions, CysLTs are generated under hypoxic and ischemic conditions causing augmentation of vascular reactivity and propagation of coronary vasoconstriction [49,65]. Thus, CysLTs lead to noteworthy coronary hemodynamic changes. For example, administration of LTD4 in patients with significant coronary stenosis leads to an augmentation of coronary resistance measured by coronary angiogram [66]. A previous in vitro study demonstrated that LTD4- and LTC4-induced coronary vasospasm during angiography was inhibited by MK [66]. It has been shown that the sensitivity of CysLTs to induce coronary vasospasm was increased due to the release of binding molecules, which increase the binding of CysLTs to the coronary atherosclerotic lesions [67]. Moreover, CysLT1Rs expression is increased in response to the released pro-inflammatory cytokines from coronary atherosclerotic lesions [67]. Interestingly, CysLT1R expression is higher than CysLT2R in the carotid and coronary intima [68]. In an experimental study, CysLTs promote the release of pro-inflammatory cytokines with induction expression of adhesion molecules [68].

These findings proposed that CysLTs play a critical role in the pathogenesis of ACS, and the use of CysLT1R antagonists such as MK might be of value in attenuating the severity of ACS (Figure 3).

## 6. Montelukast and Myocardial Infarction

It has been shown that LTs are involved in the pathogenesis of myocardial infarction. Additionally, genetic variation in 5-LO increases the risk of myocardial infarction in both animals and humans [32]. Production of CysLTs is augmented in ischemic-reperfusion injury following the development of myocardial infarction [32]. Different experimental studies illustrated that levels of CysLTs are increased after acute myocardial infarction. CysLTs induce coronary vascular resistance and increase infarct size and coronary endothelial dysfunction [69,70].

During the development of myocardial infarction, several inflammatory cells invade myocardial tissues that release LTs, which also recruit inflammatory cells and induce vascular smooth contractility, the proliferation of smooth muscle cells, and the increase of vascular permeability [71]. Gross et al.’s experimental study illustrated that production of LTs were augmented due to over-expression of 5-LO, which increases the release of pro-inflammatory cytokines [72]. A cohort study illustrated that overexpression of 5-LO increases the risk for the development of myocardial infarction [73]. Therefore, MK via inhibition of CysLT1R can reduce the severity of myocardial ischemia by inhibiting apoptosis and the production of reactive oxygen species (ROS) (Figure 3) [74].

Moreover, LTD4 plays an integral role in the development of myocardial ischemia through the induction of coronary constriction. LTD4 has been reported to be a potent coronary vasoconstrictor in the isolated heart [75]. Surprisingly, zafirlukast was shown to be ineffective against experimental myocardial ischemia since myocardial ischemia is mainly mediated by CysLT2R [76]. Notably, activation of CysLT2R results in progressive myocardial ischemia with more inflammatory changes [76]. In addition, upregulation of endothelial CysLT2R increases the risk of myocardial ischemia and apoptosis [77].

Moreover, GPR17, which is a receptor for LTD4 and LTC4, increases the risk of cardiac ischemia through interaction with cardiac CysLT1R [78]. Inhibition of GPR17 by MK reduces myocardial ischemia and fibrosis [79]. Furthermore, urinary levels of CysLTs were increased following myocardial infarction and coronary bypass surgery [79]. Another study found that rupture of coronary plaque was associated with increased urinary LTs in patients with ACS [80]. However, urinary LTs as a potential biomarker of plaque rupture or inflammatory changes during myocardial infarction need to be elucidated through further study. Many studies observed that inhibition of the LT pathway increases recovery following myocardial infarction [81,82].

Despite these findings, there are controversial studies concerning the protective effects of CysLT1R antagonists in myocardial ischemia. For example, Shekher et al. found that CysLT1R antagonists did not alter infarct size and reperfusion injury following myocardial infarction [83]. Further, coronary ligation with the development of myocardial infarction and ischemic reperfusion injury in experimental mice was not ameliorated by 5-LO inhibitors or CysLT1R antagonists [77]. However, CysLT1R antagonists may produce neutral effects [84]. Genetic variation in LTA4 leads to ethnic risk factors for the development of myocardial infarction [85]. Recently, Hori et al.’s upregulation of LTB4 receptors exacerbated inflammatory changes following myocardial infarction [86].

A systematic review involving 28 studies illustrated that CysLT1R antagonists reduce the risk of myocardial infarction, ischemic stroke, and other cardiovascular complications [87]. In addition, CysLT1R antagonists could be effective for the secondary prevention of cardiovascular diseases. The reduction of recurrent myocardial infarction in male subjects on MK therapy was significant as compared to male subjects not on MK treatment (HR = 0.65, 95% CI = 0.43–0.99) in a three-year follow-up study [87]. These findings suggest that MK may affect the expression and activity of CysLT2R, which is chiefly engaged with myocardial ischemia.

Moreover, MK attenuates lipopolysaccharide (LPS)-induced myocardial injury in a dose-dependent manner [88]. Herein, MK could have cardioprotective effects against endotoxemia-induced inflammatory changes during acute myocardial injury. This effect of MK can be attributed to its anti-inflammatory and antioxidant effects [88]. A single-center, prospective, observational study showed that patients with previous myocardial infarction had higher urinary LTs as compared with unstable angina [89].

## 7. Effects of Montelukast on the Inflammatory Signaling Pathway in Acute Coronary Syndrome

Different inflammatory signaling pathways are activated during the development of ACS that correlated with the severity of ACS. Mitogen-activated protein kinase (MAPK) is activated during ACS [90]. MAPK family includes extracellular signal-regulated kinase (ERK), c-Jun N-terminal kinase (JNK), and p38. MAPK family and toll-like receptor 4 (TLR4) are increased in patients with ACS compared to the controls [91]. Indolfi et al. confirmed that p38MAPK was increased in the T lymphocytes in patients with ACS [92]. In a comparative study, p38MAPK activities in T lymphocytes were not significant in patients with unstable angina compared to the healthy controls. However, it was higher in patients with ST-elevation myocardial infarction [92]. These findings confirmed that p38MAPK could be a potential biomarker to differentiate subtypes of ACS.

Likewise, nod-like receptor pyrin 3 (NLRP3) inflammasome is an inflammatory signaling pathway involved in the release of pro-inflammatory cytokines with the induction of pyroptosis [19]. It has been shown that NLRP3 inflammasome is activated in ACS mainly in acute myocardial infarction, causing hyperinflammation and cardiac fibrosis [93]. Mauro et al. revealed that the NLRP3 inflammasome is highly activated in acute myocardial infarction [94]. NLRP3 inflammasome senses intracellular signaling during tissue ischemia and provokes immune response during acute myocardial infarction [94]. In addition, coronary ischemia induces activation of NLRP3 inflammasome through ROS [95]. Activated NLRP3 inflammasome in ACS triggers inflammatory changes and cardiac dysfunction. Moreover, the mechanistic target of the rapamycin (mTOR) signaling pathway is activated during heart failure following acute myocardial infarction through the autophagic process [96]. Likewise, advanced glycation end-products (AGEPs) are triggered during ACS and contribute to the development of post-myocardial remodeling and dysfunction [97].

Therefore, targeting p38MAPK, NLRP3 inflammasome, mTOR, and AGEPs may reduce the risk of complications in patients with ACS (Figure 3). It has been illustrated that MK can inhibit both p38MAPK and NLRP3 inflammasome in different studies. It has been reported that MK inhibits microglial activation through suppression of p38MAPK signaling [98]. Further, Zhou et al. demonstrated that MK attenuated neuropathic pain by inhibiting p38MAPK and NF-κB in a rat model [99]. Updated studies showed that MK alleviates hepatotoxicity by inhibiting the NLRP3 inflammasome signaling pathway in an animal model [100,101]. Furthermore, MK and other CysLT1R antagonists have abilities to block the activation of mTOR and AGEPs in various experimental studies [102,103].

These verdicts suggest that MK may reduce the severity of ACS by reducing the activity of p38MAPK, NLRP3 inflammasome, mTOR, and AGEPs. This pleiotropic effect of MK may not be mediated by blocking CysLT1R; thus, extensive researches are warranted in this regard.

Taken together, MK could be an effective novel therapy in the management of ACS through inhibition of pro-inflammatory CysLT1R and modulation of inflammasome signaling pathways. Experimental, preclinical, and clinical studies are recommended to confirm the potential therapeutic of MK in the management of ACS.

## 8. Montelukast and Thrombosis in Acute Coronary Syndrome

Thrombosis plays an integral role in the pathogenesis of ACS since disruption and rupture of coronary atherosclerotic plaque expose subendothelial collagen with the release of tissue factors and pro-coagulant molecules, which activate platelet activations and fibrin formation [104]. These changes induce endothelial dysfunction and immunothrombosis with the development of coronary obstruction and subsequent ischemic-reperfusion injury in ACS [104,105]. Platelet activations trigger atheromatous complications after rupture of the coronary atheromatous plaque [104,105].

Moreover, inflammation through activation of monocytes, macrophages, and mast and T cells contributes to the pathogenesis of ACS via rupture of atherosclerotic plaque and release of pro-inflammatory cytokines [106]. Pro-inflammatory cytokines induce the expression of endothelial adhesion molecules with succeeding thrombus formation [106,107,108].

It has been shown through in silico and vitro experiments that MK had an antithrombotic effect by inhibiting factor XI (FXIa) [109]. In vivo study has also confirmed the inhibitory effect of MK on the activation of FXIa [110]. Houard and colleagues illustrated that expression of 5-LO and LTB4 are increased in the intramural thrombus [110]. Thus, LTs are engaged with the propagation of coronary thrombosis. A previous study revealed that peptido-LTs are potent agonists of clotting factors including the von Willebrand factor with induction expression of P-selectin from endothelial cells [111]. Notably, LTC4 induces the release of thromboxane A2, IL-33, and high-mobility group box 1 (HMGB1) from activated platelets, causing immunothrombosis [112,113,114]. Tang et al. observed that stimulation of GRP91 increases platelet aggregation and release of LT4 [115]. Therefore, CysLT1R inhibitors such as MK may reduce platelet activation and aggregation during the propagation of ACS and thrombotic events.

Taken together, MK has the ability to attenuate thrombotic events by inhibiting platelet activation and clotting factors, which are activated during the development of ACS (Figure 3).

The present review had many limitations, including a paucity of clinical studies that evaluate the clinical effectiveness of MK in patients with ACS. Moreover, sequential levels of LTs were not assessed in most of the clinical studies to find the association with severity and recovery.

## 9. Conclusions

ACS is associated with activation of LT pathway with subsequent releases of various LTs, including LTB4, LTC4, and LTD4, which cause inflammatory changes and the induction of immunothrombosis. LTs through CysLTRs induce activation of platelets and clotting factors with succeeding coronary thrombosis. CysLTR antagonists such as MK may reduce risk development of ACS and associated complications through suppression activation of platelet and clotting factors. Taken together, MK could be an effective agent in reducing severity of ACS and associated complications. Experimental and clinical studies are warranted in this regard.

## Data Availability

Data sharing not applicable.
